# Development of phytotherapeutic nanoformulation containing *Gypsophila eriocalyx* and its evaluation as a candidate formulation for osteoporosis treatment on human bone marrow stem cells

**DOI:** 10.1002/pca.3440

**Published:** 2024-08-26

**Authors:** Sibel Kaymak, Ozan Baris Kurtur, Bahar Gok, Yasemin Budama‐Kilinc, Serda Kecel‐Gunduz, Ebru Özdemir Nath, Murat Kartal

**Affiliations:** ^1^ Graduate School of Natural and Applied Science, Department of Bioengineering Yildiz Technical University Istanbul Turkey; ^2^ Department of Traditional, Complementary and Integrative Medicine, Biotherapeutic Products Research and Development Program Ankara Yildirim Beyazit University Ankara Turkey; ^3^ Faculty of Chemical and Metallurgical Engineering, Department of Bioengineering Yildiz Technical University Istanbul Turkey; ^4^ Health Biotechnology Joint Research and Application Center of Excellence Istanbul Turkey; ^5^ Faculty of Science, Physics Department Istanbul, Turkiye Istanbul University Istanbul Turkey; ^6^ Department of Pharmaceutical Botany, Faculty of Pharmacy Altınbaş University Istanbul Turkey; ^7^ Altınbaş University Natural Products Research and Development Center (DÜAGEM), Altınbaş University Istanbul Turkey; ^8^ Faculty of Pharmacy, Pharmacognosy Department Bezmialem Vakif University Istanbul Turkey

**Keywords:** chitosan nanoparticles, medicinal plant, molecular docking, osteoporosis, stem cell

## Abstract

**Introduction:**

Osteoporosis, one of the common bone diseases, manifests itself as a decrease in bone mass. Recently, the use of medicinal plants in the search for effective and low‐toxicity therapeutics for the prevention or treatment of osteoporosis has become a trending topic.

**Objective:**

In this study, we aim to prepare a controlled drug carrier system loaded with *Gypsophila eriocalyx* to determine its potential for anti‐osteoporosis applications.

**Methods:**

*Gypsophila eriocalyx* extract (GEE) was prepared, and components were determined. The molecular interactions of the components with Cathepsin K (CatK), which is used as a target in drug development against osteoporosis, were revealed by *in silico* molecular docking and MD methods. ADMET profiles were also examined. GEE‐loaded chitosan nanoparticles (CNPs) were synthesized. The nanoparticles' morphology, encapsulation efficiency, loading capacity, release profile, average size, polydispersity index, and zeta potentials were determined. The cytotoxic effects of GEE and GEE‐loaded CNPs on the L929 and osteogenic proliferation profiles on human bone marrow stem cells (hBMC) were examined.

**Results:**

The MD analysis revealed no breaks or atomic changes in the dynamic system, and the docking analysis confirmed the continued interaction of identical residues. It was determined that the GEE‐loaded CNP formulation was produced successfully, had no toxic effect on the L929, and had an osteogenic proliferation effect on hBMC.

**Conclusion:**

In line with the *in vitro* and *in silico* results obtained, it was evaluated that GEE‐loaded CNPs can be used as a controlled drug release system as a candidate formulation with phytotherapeutic properties for osteoporosis treatment.q1

## Abstract 2

In this study, Gypsophila eriocalyx extract (GEE) loaded CNPs were synthesized and their potential use in osteoporosis treatment was investigated. The GEE‐loaded CNPs were non‐toxic to L929, exhibited a long‐term and controlled release profile, and enhanced osteogenic proliferation in hBMC. GEE phytochemicals exhibited hydrophobic and electrostatic interactions, and neither the phytochemical nor the receptor displayed any signs of atomic or bond breaking, according to in silico analysis. Therefore, GEE‐loaded CNPs may be a useful approach in the treatment of osteoporosis.

## 1 INTRODUCTION

Osteoporosis is a common disease characterized by decreased bone mass and the deterioration of bone tissue and microarchitecture. These changes in the bone matrix result in reduced bone strength and an increased risk of fractures.^1^ Osteoporosis progresses painlessly, and if left untreated, it can result in serious fractures in the spine, wrist, and hip, causing disability and death, particularly among the elderly.^2^ It is estimated that more than 200 million people worldwide are affected by osteoporosis, with the prevalence increasing with age.^3^ Moreover, these fractures due to osteoporosis are thought to affect 20% of men and 33% of women.^4^ Despite numerous ongoing studies, there is still no definitive treatment, and current therapies are mostly limited to resorptive drugs and anabolic agents. Most clinically used anti‐osteoporotic drugs are administered systemically, which can have side effects in nonskeletal tissues.^5^ Therefore, searching for effective treatment sources with a lower side effects has become crucial.

Medicinal plants are a rich source of compounds that can be used in pharmacopeial, non‐pharmacopeial, or synthetic medications.^6^ Many plants are currently being used or researched for their potential to treat diseases because of their phytochemical content. Moreover, these plants are an essential resource for developing new drugs to treat osteoporosis.^7^ For example, the effects of saponins extracted from the *Panax notoginseng* plant on radiation‐induced osteoporotic mice were examined, and the results revealed that the plant has an anti‐osteoporotic impact.^8^ In another study, the anti‐osteoclastogenic activities of saponins that are obtained from *Mussaenda pubescens* were examined, and as a result, saponins significantly reduced the activities of bone‐resorptive cells called osteoclasts.^9^

Gypsophila species are promising for treating diseases due to their antitumor, antimicrobial, hepatoprotective, and antidiabetic properties. Additionally, numerous studies have demonstrated that Gypsophila species exhibit anti‐osteoporotic effects.^10^, ^11^ Gypsophila species are promising for treating diseases due to their antitumor, antimicrobial, hepatoprotective, and antidiabetic properties.^12^ The endemic species *Gypsophila eriocalyx*, which belongs to the genus Gypsophila, has excellent pharmacological potential because of its rich phytochemical content.^13^ This endemic plant species contains high amounts of triterpenoid saponin, also known as gypsogenin and various flavonoids such as quercetin 3‐rutinoside (rutin) and rosmarinic acid.^14^ Moreover, it has been suggested that *G. eriocalyx* is a strong therapeutic plant candidate that can be used as an alternative in treating progressive diseases such as diabetes, cancer and Alzheimer's due to oxidative damage.^15^ However, studies to evaluate the potential use of *G. eriocalyx* in the treatment of osteoporosis are lacking in the literature.

Nanoformulations have become dosage systems that researchers have turned to in order to reduce the disadvantages of currently used treatments in dealing with osteoporosis and to develop both innovative and alternative treatment approaches.^16^ One such innovative approach is the development of controlled drug release systems that ensure that drugs are delivered to the desired body area in the required amounts and for an extended duration.^17^ Biopolymer‐based nanoparticles are promising for controlled drug systems because they have low toxicity, a high surface area, and are biocompatible. Biopolymers are effective drug delivery carriers because of their high stability, loading capacity, and adjustable properties.^18^ In this context, chitosan, a natural biopolymer derived from chitin, is gaining attention in nanocarrier systems for its mucoadhesive, biocompatible, non‐toxic, biodegradable, and specific delivery properties.^19^ Moreover, chitosan‐based nanoparticles have gained popularity as drug carriers for the treatment of osteoporosis due to their small size, high encapsulation efficiency, loading capacity, and ability to combine with various molecules, including drugs or plant components.^2^ In a study, risedronate‐functionalized chitosan nanoparticles were developed to treat osteoporosis and were highly influential in stimulating bone formation in osteoporotic rats.^20^ It has been reported that transdermal gel developed with risedronate chitosan nanoparticles has the potential to be used as an alternative in the treatment of osteoporosis.^21^ Moreover, nano‐chitosan conjugates developed with Shilajit aqueous extract significantly improved osteoporosis‐related markers and were reported to be more effective than the extract.^22^ It has been reported that *Lepidium sativum* L extract‐loaded chitosan nanoparticles regulate biochemical bone indices, serum calcium and phosphorus levels, miR23a and miR‐142‐3p expressions in osteoporotic rats, and thus have the potential to be used as a therapeutic agent in the treatment of osteoporosis.^23^ However, despite the medicinal potential and the effectiveness of *G. eriocalyx*, its chitosan‐based controlled release system for osteoporosis has not been investigated.

In this study, *G. eriocalyx* extract (GEE) was obtained from *G. eriocalyx* roots using the decoction method, and its saponin and rutin contents were determined by the double solvent extraction gravimetric method and HPLC, respectively. In addition, GEE‐loaded CNPs were synthesized and characterized, and their release profiles, cytotoxicity, and *in silico* anti‐osteoporosis effects were evaluated. The average particle size, zeta potential, and polydispersity index (PdI) of the particles were determined using dynamic light scattering. Chemical interactions between GEE and the chitosan polymer were analyzed using ATR‐FTIR. The loading capacity, *in vitro* release profile, and encapsulation efficiency of the nanoparticles were determined using a UV–Vis spectrophotometer. MTT assays were performed to assess the synthesized nanoparticles' cytotoxicity and osteogenic proliferation profiles. Finally, the anti‐osteoporosis effects of some natural bioactive molecules from *G. eriocalyx* against Cathepsin K (CatK), a key target for anti‐osteoporosis drug development, were investigated using virtual screening, molecular docking, molecular dynamics, and ADME (Absorption, Distribution, Metabolism, Excretion) prediction approaches.

## 2 MATERIALS AND METHODS

### 2.1 Materials

Chitosan (75%–85% deacetylated, low molecular weight, CAS no. 448869) and sodium tripolyphosphate (TPP, CAS no. 72061) were purchased from Sigma Aldrich (St. Louis, MO, USA). Acetic acid (CAS no. 1.00056.2500) was purchased from Merck. Ethanol (CAS no. 920.026.2500) was purchased from ISOLAB. Human bone marrow stem cell (ATCC®) was a kind gift from Labcell. Fetal Bovine Serum (FBS, CAS no. BI04‐007‐1A) was purchased from Biological Industries. Dulbecco's Modified Eagle Medium (DMEM, CAS no. 11995065) and Trypsin‐EDTA (CAS no. 25200‐056) were purchased from Gibco™. Dimethyl sulfoxide (DMSO, CAS no. A3672,0250) was purchased from Panreac & Applichem (ITW Reagents). Thiazolyl Blue Tetrazolium Bromide (MTT, CAS no. M5655) was purchased from Sigma Aldrich. All the chemicals and solvents used in the study were of analytical grade.

### 2.2 Methods

#### 2.2.1 Identification of *G. eriocalyx* and preparation of *G. eriocalyx* extract

The roots of *G. eriocalyx* were collected from the Iskilip (Corum, Turkey) in July, at a latitude and longitude of 40°43′53.5″N 34°27′14.7″E. The plant materials were identified by Asst. Prof. Ebru Ozdemir Nath (Department of Pharmaceutical Botany, Faculty of Pharmacy, Altınbaş University, Istanbul, Turkey), and they were deposited with the number “1,055” in the Herbarium of Altınbaş University Faculty of Pharmacy (HERA).

GEE was obtained by the decoction method. Briefly, 2 g of ground dried roots of *G. eriocalyx* were taken and placed in 100 mL of distilled water. The mixture was stirred at 250 rpm and 70°C, and after the desired amount of extraction, it was filtered through 0.45‐μm filter paper. The resulting solution was lyophilized, and the product was stored at +4°C.

#### 2.2.2 Determination of GEE composition

GEE composition was determined by total saponin and rutin analysis. The saponin content of the GEE was determined by double solvent extraction gravimetric method. The 2 g of the GEE was mixed with 50 mL of 20% aqueous ethanol solution. The mixture was heated with periodic agitation in a water bath for 90 min at 55°C. It was filtered through Whatman filter paper. The residue was extracted with 50 mL of 20% ethanol, and both extracts were pooled together. The combined extract was reduced to about 40 mL at 90°C and transferred to a separating funnel where 40 mL of diethyl ether was added and shaken vigorously. Separation was done by partition, during which the ether layer was discarded and the aqueous layer was reserved. Re‐extraction by partition was done repeatedly until the aqueous layer became clear in color. The saponins were extracted with 60 mL of normal butanol. The combined extracts were washed twice with 10 mL of a 5% aqueous NaCl solution and evaporated to dryness in a pre‐weighed evaporating dish. It was dried at 60°C in the oven and reweighed. The experiment was repeated three times to get an average. Equation (1) was used to determine the total saponin percentage. In this equation, W1 represents the weight of the evaporating dish, and W2 represents the Weight of the dish and GEE.

(1) $$\text{Saponins}\;\left(\%\right)=\frac{W_{1}-W_{2}}{\text{Weight of}\;\mathrm{GEE}}\;x\;100$$

Moreover, rutin was quantified by high‐performance liquid chromatography (HPLC) on a Shimadzu LC2050C Prominence‐i (Japan) system at UV detection of 360 nm. Rutin peak was identified based on retention time (RT) matched with the corresponding reference standard. RP‐HPLC analysis was performed by gradient elution with a low‐pressure gradient using 0.1% phosphoric acid in water: acetonitrile as a mobile phase with a flow rate of 0.5 mL/min. The separation was done at 25°C using a GL Science Intersil‐C18 column (250 mm × 4.6 mm × 5 μm) as the stationary phase and the detection wavelength at 360 nm. The mobile phase consisted of water containing 0.1% formic acid (A) and acetonitrile (B). The composition of the mobile phase was from 0% to 30% (B) for 0–30 min, 30%(B) for 30–40 min, 30%–50% (B) for 40–70 min, 50%–30% (B) for 70–75 min, 30%–0% (B) for 75–85 min, and it was held for 5 min and then re‐equilibrated to 0% (B) until the end of the analysis. The flow rate was 0.5 ml/min, and the injection volume was 20 μL. The detection wavelengths of all standards and samples were in the UV at 360 nm.

For the preparation of standard solutions 10 mg of the pure standard substance of rutin was accurately weighted, transferred to a 1000‐mL volumetric flask and made up to 1000 mL with methanol. Then, 0.01, 0.005, 0.002, 0.001, 0.0005 μg/mL solutions were prepared by stepwise dilution. These standard solutions were stored at 4°C. For the sample preparation, 2 g of the dried GEE extract was immersed in 10 mL of 80% methanol for 24 h, and the resulting solution was treated using ultrasound‐assisted extraction procedure two times, each time for 20 min. The combined extract was evaporated to dryness in a rotary evaporator at 65°C, and the residue was reconstituted with 100 ml of mobile phase, filtered through a 0.45 syringe filter and injected into HPLC system.

#### 2.2.3 In silico analysis

Within the scope of in silico analyses, molecular docking analyses, molecular dynamics simulations and evaluation of drug likeliness properties and pharmacokinetics (ADMET) studies were carried out.

##### Molecular docking analyses

The molecular docking method is a target‐based analysis in silico that analyzes the alignment and orientation of molecules to the binding sites on macromolecule targets. Making predictions of molecular interactions is a preliminary step in determining potential drug behaviors toward the target. Molecular docking analysis saves both time and resources in drug development studies. *G. eriocalyx*, an endemic species of the Caryophyllaceae family known for its rich herbal ingredients, contains many triterpenoid saponins and various flavonoids in its structure.^24^ Saponins, the main components of Gypsophila species, are evaluated as terpenoids in secondary metabolite groups according to their chemical structure. Saponins are naturally occurring bioorganic molecules with 27 or 30 carbon, high molecular weight, aglycone nuclei, and one or more sugar moieties containing at least 6 or 12 carbon atoms, respectively. The saponins seen in the Caryophyllaceae family are mostly gypsogenin, gypsogenic acid, or quillaic acid.^25^ Of the saponins belonging to the family, 46% are defined as gypsogenin, 33% as quillaic acid and 31% as gypsogenic acid. Especially in species of the genus Gypsophila, gypsogenin is the most abundant component in their structures, with a rate of 75%.^26^ As a result of the studies carried out in the aqueous solutions of *G. eriocalyx* in the literature, quercetin 3‐rutinoside and rosmarinic acid components have been reported in addition to the saponin substance known as the main component in Gypsophila species.^26^ Rutin, rosmarinic acid, and p‐coumaric acid are among the phenolic compounds whose amounts are stated in different extracts of Gypsophila taxa.^26^ In this study, gypsogenin (Pubchem ID: 92825),^27^ rutin (Pubchem ID: 5280805)^28^ and rosmarinic acid (Pubchem ID: 5281792),^29^ classified as saponin and phenolic compounds were preferred as ligands from *G. eriocalyx* to investigate the anti‐osteoporosis effects against the CatK receptor using the molecular docking analysis method.

The molecular structures of gypsogenin, rutin, and rosmarinic acid, which are the most dominant phytochemicals in *G. eriocalyx*, were downloaded from the PubChem site and were introduced to the Maestro 11.4 Glide^30^ module of the Schrödinger program. The Glide SP module of Maestro version 11.4 from Schrodinger Software^30^, ^31^ was performed for molecular docking analysis. All ligands were introduced to the Ligand builder panel, and then the optimization process was performed using the LigPrep module. The OPLS3^32^ force field was used for energy minimization process. After energy minimization, all stereoisomers of each ligand in different geometries (32 conformations) at neutral pH were also obtained for docking analysis.

CatK, whose main function is to mediate bone resorption, is one of the proteases in the lysosomal cysteine protease family. Osteoclasts mostly secrete CatK to break down collagen and other matrix proteins during bone resorption.^33^ It catalyzes the degradation of Type 1 collagen. A few CatK inhibitors have been developed but have yet to be used due to a lack of selectivity, side effects, or drug interactions.^34^ It is an attractive target for anti‐osteoporosis drug development and is studied by pharmaceutical companies. CatK was selected as a receptor for docking analysis.

CatK^33^, ^35^ crystal structure with 2.20 Å resolution and ∼215 residues was selected for the receptor. CatK (pdbID: 1ATK)^35^ was pulled from the protein database, and the arranged form was obtained with the help of the SWISS‐MODEL^36^ server.

Using the “receptor preparation wizard” in the Glide module of the Schrödinger Maestro Program, all water and ion structures in the receptor structure were removed, polar hydrogens were added, bond patterns were determined, and charges were defined with PROPKA^37^ at neutral pH (7.0). The CatK receptor structure was also optimized and minimized^38^ using the OPLS3 force field after all preprocessing. By creating 3D grid boxes centered on the center of gravity of each ligand, all residues containing thiol and hydroxyl groups in the binding region of the receptor were identified, and the docking of each ligand to the receptor was analyzed, respectively.

##### Molecular dynamics simulations

CatK is among the most critical targets for anti‐osteoporosis drug development. In a longer‐term dynamic system, MD simulations were performed for 50 ns using Desmond simulation package of the Schrödinger program^39^, ^40^ to analyze the interaction between CatK and the most dominant phytochemicals in *G. eriocalyx* (gypsogenin, rutin, and rosmarinic acid).

All systems were solved using a cubic box containing the TIP3P water molecule model, and Na^+^ and Cl^−^ ions were added to neutralize the net charge of the systems. Predicting a pressure of 1 bar and a temperature of 300 K, NPT ensembles were applied to all systems, and they were subjected to final MD simulations for 50 ns. After obtaining the analysis of MD trajectories using the tools provided by the Desmond simulation package of the Schrödinger program, the root‐mean‐square deviation (RMSD) analysis, the root‐mean‐square fluctuations (RMSF) plots of C_α_ atoms, side chain, as well as receptor‐ligand contacts and interactions were also performed.

##### Evaluation of drug likeliness properties and pharmacokinetics (ADMET)

The pharmacokinetics that is known as absorption, distribution, metabolism, and excretion (ADMET) properties, drug‐likeness, and oral bioavailability profiles of *G. eriocalyx* phytochemicals were predicted using the SwissADME web tool.^41^, ^42^

#### 2.2.4 Synthesis of GEE‐loaded CNPs

GEE‐loaded CNPs were synthesized via the ionic gelation method using our previous studies with slight modifications.^43^, ^44^ This method is based on the interaction between sodium tripolyphosphate (TPP), a polyanionic agent, and chitosan, a polycationic agent.^45^ In this context, 20 mg of low molecular weight chitosan was weighed and dissolved in 100 mL of water containing 0.3% acetic acid solution and then stirred continuously on a magnetic stirrer for 24 h. To prepare the TPP solution, 50 mg of TPP was dissolved in 100 mL of distilled water. Then, 2 mg of lyophilized GEE was dissolved in a certain amount of TPP. As a final step, chitosan and TPP solutions were mixed at a v/v ratio of 5:2 at 14 mL final volume and stirred for 15 min (850 rpm). Finally, the nanoparticle solution was filtered through 0.45 μm filter paper and was dried in a freeze‐dryer (Biobase, Shandong, China). Blank CNPs were synthesized by the same method without adding GEE.

#### 2.2.5 Characterization methods of CNPs

The physicochemical characterization of the obtained nanoparticles was carried out with different spectroscopic and imaging methods, such as UV–Vis, dynamic light scattering, ATR‐FTIR, and TEM.

The Malvern Zetasizer Nano ZS instrument that has a 4.0‐mV He‐Ne laser (633 nm) and operates at 25°C was used for PdI, average size, and zeta potential analyses. FTIR and ATR analyses were performed to examine the chemical interactions between GEE and chitosan polymer. For GEE, GEE‐loaded CNPs, and blank CNPs, data were obtained by attaching and using ATR measurement head to Jasco 6300 FTIR spectrometer device, with ATR spectrum in the range of 2 cm^−1^ resolution and 4000–400 cm^−1^. Morphological analysis of GEE‐loaded CNPs was performed using TEM (JEOL JEM‐1400 Plus). TEM images were obtained by operating at 80 kV voltage. The 3 mg of dry sample was dispersed in isopropyl alcohol. The 1 μL of the dispersed sample was dropped onto a carbon film‐supported copper grid and analyzed after the solvent had evaporated. Samples at different concentrations (6.75, 12.5, 25, 50, 100, 200, 400 μg/mL) were prepared by diluting the 1 mg/mL stock solution prepared with the GEE in order to prepare the calibration curve. Then, absorbance values of samples at different concentrations were measured with a UV–Vis Spectrophotometer to obtain the calibration curve. This curve was used to determine the encapsulation efficiency, loading capacity and *in vitro* release profile of GEE‐loaded CNPs. The calibration curve of GEE was used to determine encapsulation efficiency (EE) and loading capacity (LC). Equation (2) was used to determine the amount of GEE encapsulated.

(2) $$\text{Encapsulation Efficiency}\;\left(\%\right)=\frac{\text{Total}\;\mathrm{GEE}\;\text{Amount}-\text{Free}\;\mathrm{GEE}}{\text{Total}\;\mathrm{GEE}\;\text{Amount}}\times 100$$

Further, the solution of GEE‐loaded CNPs were lyophilized and weighed. The loading capacity of GEE loaded CNPs was calculated with Equation (3).

(3) $$\text{Loading Capacity}\;\left(\%\right)=\frac{\text{Encapsulated}\;\mathrm{GEE}\;\text{Amount}}{\text{Total Nanoparticle Weight}}\times 100$$

To examine the release of GEE from GEE‐loaded CNPs, *in vitro* release experiments were performed by imitating the body environment. First, a pre‐determined amount of lyophilized nanoparticles were dissolved in 2 mL distilled water and put into 100 ml PBS (pH 7.4) solutions. Then, PBS solutions were placed into a shaking water bath at 37°C and 120 rpm. During the release assay, 1 mL of the release medium was taken at specific time intervals for spectrophotometric analyses. Then 1 mL of fresh PBS was added to maintain the total volume of all release systems. Release samples were measured using a UV spectrophotometer at the specific wavelength of GEE, and absorbance values were determined. Here, the blank chitosan nanoparticles were used for baseline measurement. Then, these absorbance values were used to determine concentrations through the calibration curve, and these concentrations were used to calculate the release profiles of GEE‐loaded CNPs using Equation (4).

(4) $$\text{Release}\;\left(\%\right)=\frac{\text{Released Amount of}\;\mathrm{GEE}}{\text{Total Amount of}\;\mathrm{GEE}}\times 100$$

#### 2.2.6 *In vitro* cytotoxicity of GEE‐loaded CNPs

The mouse fibroblast cell line (L929) was used to determine the cytotoxicity of GEE‐loaded CNPs. L929 cell line was cultured in DMEM medium (including 10% Fetal Bovine Serum and Penicillium‐Streptomycin) at 37°C and 5% CO_2_ level. Then, proliferated L929 cells were removed from the flask surface by trypsin. To examine the toxic effects of GEE‐loaded CNPs by MTT test, cells prepared in a medium containing 1% penicillin–streptomycin and 10% Fetal bovine serum (FBS) in DMEM‐F12 were added to microplates containing 96 wells and incubated for 24 h. Cells were seeded with GEE‐loaded CNPs at a concentration of 0.125, 0.25, 0.5, and 1 mg/mL, while the concentration for GEE was determined according to the loading capacity. Then, 40 μL of MTT solution was added to the wells and incubated for 4 h at 37°C without light. Finally, 160 μL of dimethylsulfoxide (DMSO) was added to the incubated cells, and after waiting for 30 min, the plate was measured using an ELISA reader (Biotek, EPOCH).

#### 2.2.7 *In vitro* osteogenic proliferation of GEE‐loaded CNPs

Human bone marrow stem cells (hBMC) were used to determine the contribution of GEE‐loaded CNPs to osteogenic proliferation. The cells were cultured in flasks containing DMEM supplemented with 10% FBS and 1% penicillin–streptomycin. Then cells were incubated at 37°C in 5% CO_2_ humidified for 2 days. After 90% of cell confluence, the culture was sub‐cultured by trypsin–EDTA for 5 min at 37°C. The osteogenic proliferation of GEE‐loaded CNPs was determined by performing an MTT assay with hBMCs. The hBMC cells were added to the wells with GEE‐loaded CNPs and then incubated with MTT for 4 h at 37°C. Then DMSO was added, and the plate was measured using an ELISA reader (Biotek, EPOCH).

#### 2.2.8 Statistical analysis

For MTT tests (both cytotoxicity and osteogenic proliferation), the results of the measurements (*n* = 3) obtained from the control and treatment groups were compared. Differences between group mean for the test system were evaluated with one‐way ANOVA (analysis of variance) using the IBM SPSS Statistics 21 package program. The significance level of the differences between the group mean was determined at the *p* < 0.05 level with the Tukey test according to the homogeneity of the variances. Also, mean values are presented as ±SD.

## 3 RESULTS AND DISCUSSION

### 3.1 Determination of GEE composition

Total saponin content (%) and rutin analysis in GEE were determined by double solvent extraction gravimetric method and RP‐HPLC analysis, respectively. The total saponin content of GEE was found 3.98 ± 0.05%. In addition, the calculated contents of the rutin were found as 70 μg/g in GEE extract below the limit of quantification.

### 3.2 In silico analysis results

#### 3.2.1 Molecular docking analysis results

**FIGURE 1:**
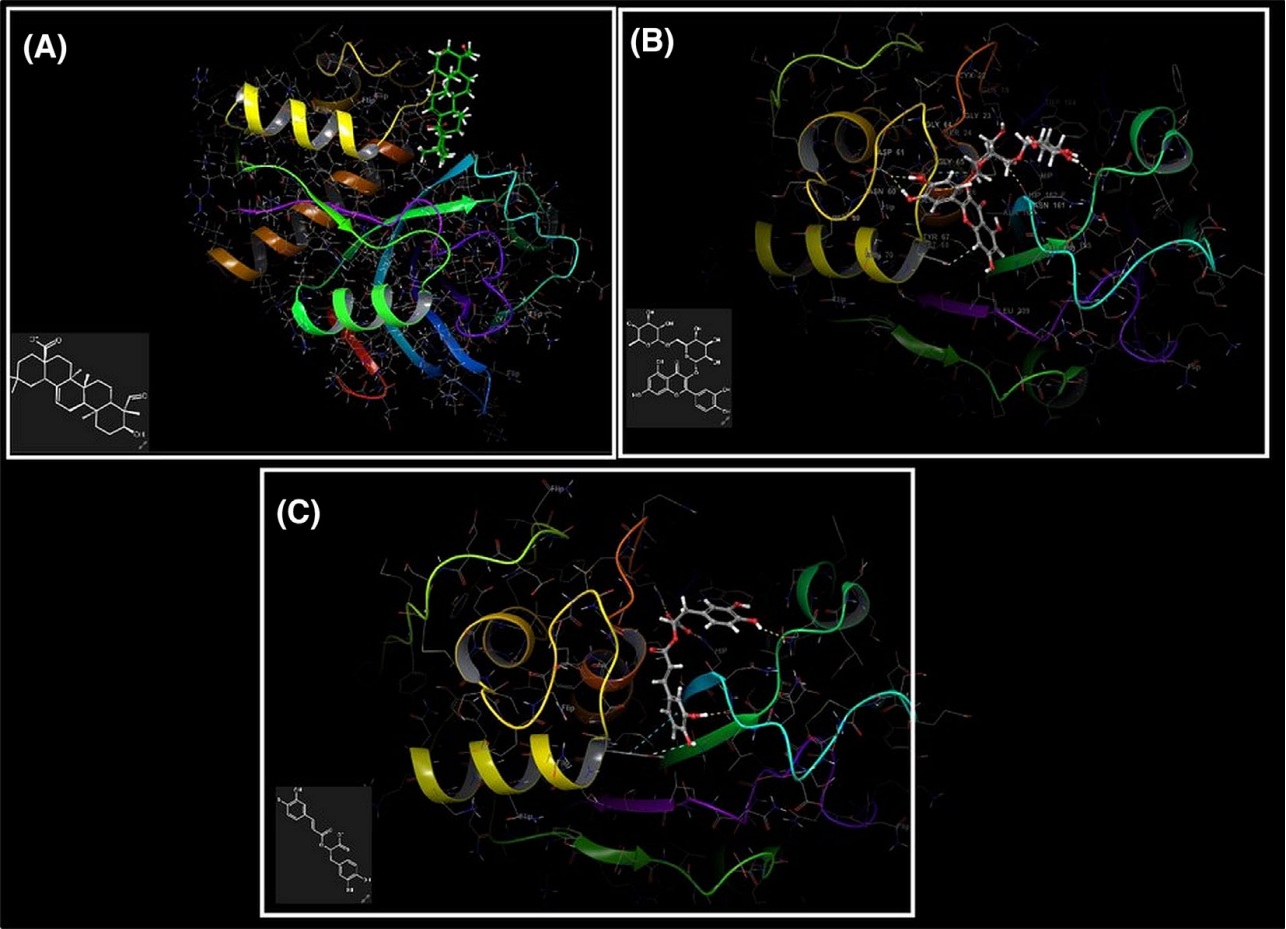
Docked 3D structures of the active ligands gypsogenin (A), rutin (B), and rosmarinic acid (C) that binded to the Cathepsin K (CatK) receptor.

By using molecular docking analysis, the docking and interaction mechanisms of gypsogenin, rutin and rosmarinic acid's molecular structures, which are the most dominant phytochemicals in *G. eriocalyx*, with CatK, which is one of the most important targets for anti‐osteoporosis drug development, were revealed. In Figure 1, docking poses of three phytochemicals with CatK receptor were seen, and at the same time, the binding energies of these docking and the interaction types with the receptor were presented in Table 1.

**TABLE 1:** Types of interaction with the Cathepsin K protein (PDB ID:1atk), one of the protease responsible for osteoporosis‐induced bone resorption, with the docking score energies of the dominant molecules in *Gypsophila eriocalyx*.

	Gypsogenin C_30_H_46_O_4_	Rutin C_27_H_30_O_16_	Rosmarinic acid C_18_H_16_O_8_
**Docking Score (Kcal/mol)**	−5.75	−8.67	−6.20
**H.Bonding interaction** **(Angstrom)**	ASP61(1.80) GLY66(1.84)	SER138(1.86) ASN161(2.18) GLY66(2.44) ASP61(1.71) ASP61(1.74) HIS162(2.71)	LEU160(1.91) GLN19(1.65) SER138(2.16)
**Salt Bridge interaction (Angstrom)**	‐	‐	HIS162(2.66)
**Aromatic H. bonding interaction (Angstrom)**	‐	TYR67(2.59) TRP26(2.71)	TYR67(2.73) TRP184(2.77)
**Pi‐Pi‐stacking interaction (Angstrom)**	‐	‐	TYR67(5.16)

**FIGURE 2:**
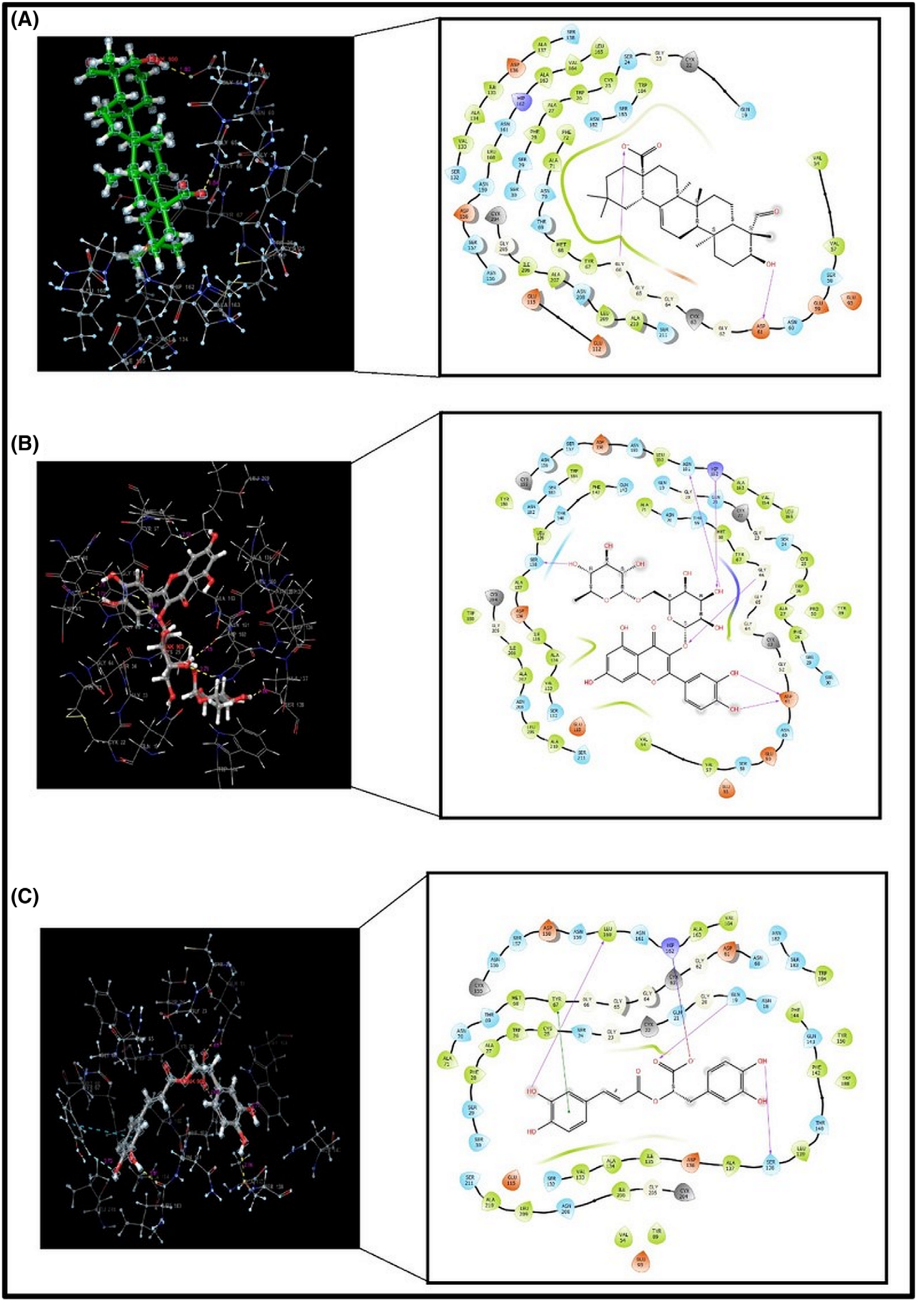
2D visualizations of the interactions of gypsogenin (A), rutin (B), and rosmarinic acid (C) active ligands binding to the active binding site of the Cathepsin K (CatK) receptor.

In addition, the receptor binding sites and interaction mechanisms of gypsogenin, rutin and rosmarinic acid molecules that binded to the active binding site of the CatK receptor are given in Figure 2. Examination of Table 1 indicated that the molecular structure that performed the most stable binding with the lowest energy to the CatK receptor belonged to rutin. The score of stable coupling between rutin and CatK receptor was given as −8.67 kcal/mol. After rutin, rosmarinic acid took second place with a docking score of −6.20 kcal/mol. Gypsogenin, on the other hand, was docked to the receptor with a docking score energy of −5.75 kcal/mol. Gypsogenin made fewer hydrogen bond interactions with the residues in the active binding site of the CatK receptor compared to rutin and rosmarinic acid. For this reason, the docking score energy was obtained larger than other phytochemicals. Gypsogenin was linked by two hydrogen bonds between residues ASP61 (1.80 Å) and GLY66 (1.84 Å) and these interactions were visualized in Figure 2A with purple arrows. Rutin, on the other hand, was clamped with a total of six hydrogen bonds with the residues SER138 (1.86 Å), ASN161 (2.18 Å), GLY66 (2.44 Å), ASP61 (1.71 Å), ASP61 (1.74 Å), and HIS162 (2.71 Å) in the active binding site of the CatK receptor. In addition to these, it has achieved stable coupling by bonding with TYR67 (2.59 Å) and TRP26 (2.71 Å) residues with aromatic hydrogen bonding interactions. The hydrogen bond interactions and the aromatic hydrogen bond interactions between the residues were given by purple arrows and blue arrows, respectively in Figure 2B. Rosmarinic acid also performed three hydrogen bond interactions with residues LEU160 (1.91 Å), GLN19 (1.65 Å), and SER138 (2.16 Å) in the active binding site of CatK receptor, while aromatic hydrogen bond interactions with residues TYR67 (2.73 Å) and TRP184 (2.77 Å) were occurred. It bonded to the receptor by performing salt bridge interaction with the HIS162 (2.66 Å) residue and also pi‐pi stacking interactions with TYR67 (5.16 Å). All these interactions are also given in Figure 2C. Hydrogen bond interactions were depicted with purple arrows, while pi‐pi stacking interaction was shown with a green line.

#### 3.2.2 Molecular dynamic (MD) analysis results

**FIGURE 3:**
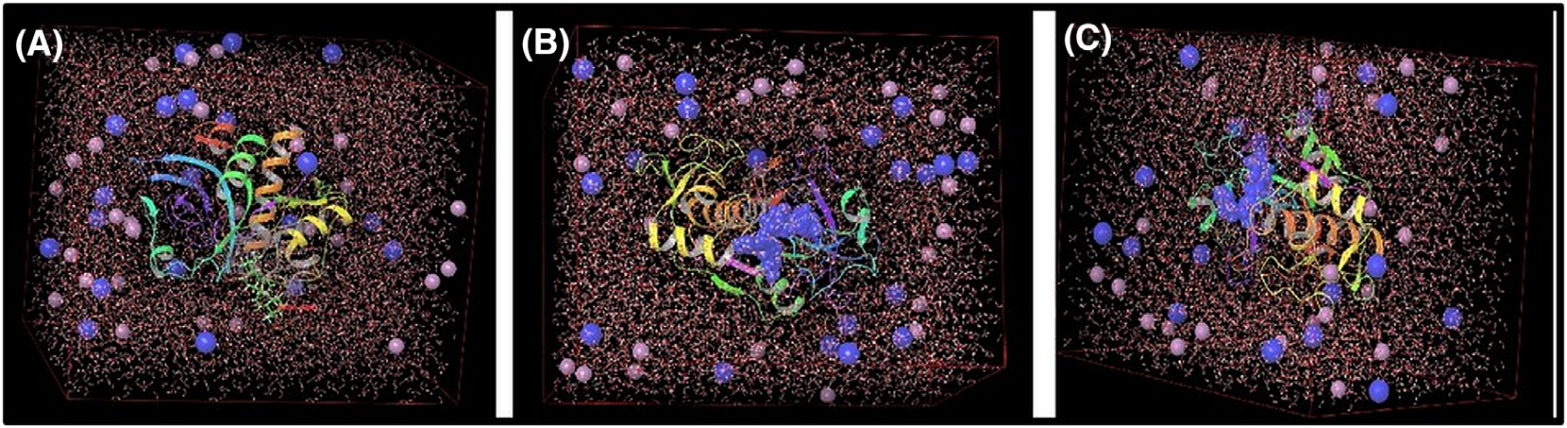
System structures prepared for MD with gypsogenin (A), rutin (B), and rosmarinic acid (C) with Cathepsin K (CatK) receptor with cartoon image with water molecules and Cl and Na ions.

By choosing the lowest energy docking poses as the starting geometry for MD simulation, the receptor interactions of all three phytochemicals in a dynamic environment for a long process were revealed for the first time with this study. For the gypsogenin molecule, MD simulation was performed for 50 ns at 300 K temperature and 1 atm pressure by adding 7052 water molecules together with CatK and 27 Cl^−^ and 20 Na^+^ atoms to neutralize the system. For the rutine molecule, 6741 water molecules along with CatK were added to the system and to neutralize the system, 27 Cl^−^ and 19 Na^+^ atoms were also added, and the system was simulated for 50 ns at 300 K temperature and 1 atm pressure. Rosmarinic acid molecule was also monitored with CatK together with 6768 water molecules and 26 Cl^−^ and 19 Na^+^ atoms at the same temperature and pressure values with the same simulation time. These three systems prepared for MD are shown in Figure 3.

**FIGURE 4:**
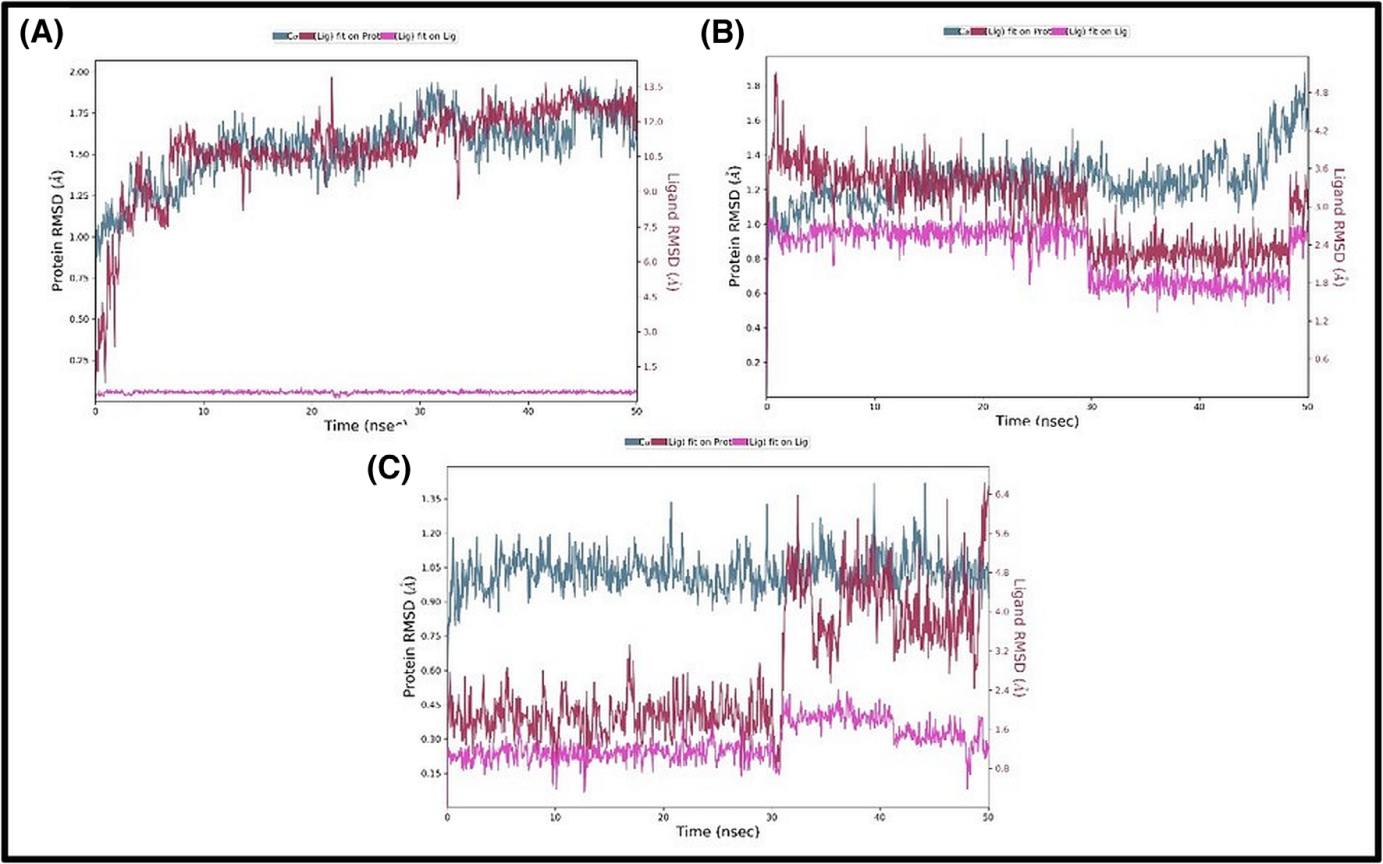
Root‐mean‐square deviation (RMSD) values for 50 ns of MD systems prepared with gypsogenin (A), rutin (B), and rosmarinic acid (C) and Cathepsin K (CatK).

As a result of 50 ns MD calculation, the trajectory values of the systems were analyzed and the RMSD values of each system were calculated one by one to analyze the stability of the system. Protein and ligand RMSD values of the systems formed with gypsogenin, rutin, and rosmarinic acid were plotted in Figure 4A–C, respectively.

**FIGURE 5:**
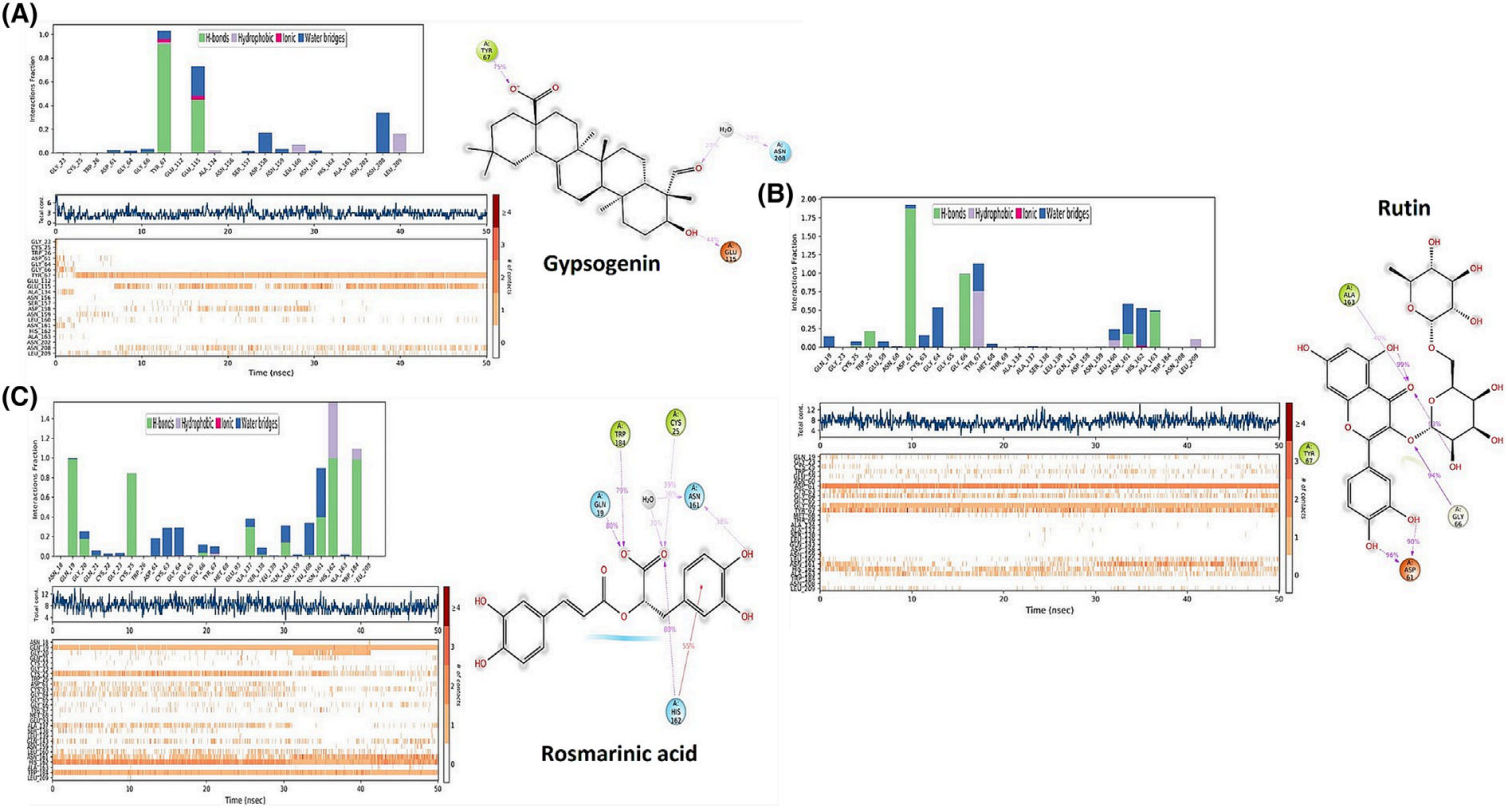
Interaction of molecules with Cathepsin K (CatK) receptor during 50 ns simulation time; (A) gypsogenin, (B) rutin, and (C) rosmarinic acid.

According to this figure, the RMSD values of the protein alpha carbon drawn with the blue line were obtained below 2 Å. The ligand RMSD value expressed by the pink colored line was also obtained below 0.5 Å for the first system. In the second system, this value was around 1 Å in the first 30 ns, while after 30 ns it decreased to around 0.6 Å. For the third system, this value remained below 0.3 Å during the first 30 ns, while it increased to 0.45 Å in the remaining period. The RMSD value of the ligands in the active binding region of the protein was visualized with a dark pink line. During the MD calculation, these RMSD values remained stable around 1.50 Å with small fluctuations. The protein and ligand RMSD values calculated for the rutine were also obtained below 2 Å in Figure 4B, and the RMSD value for the system created for rosmarinic acid was calculated at a maximum as 1.35 Å and was shown in Figure 4C. It was obtained in a stable state in these three systems, no fragmentation of atoms or breakage of bonds was observed. The interactions of the gypsogenin, rutin and rosmarinic acid molecules with CatK at 50 ns simulation time were given in Figure 5A–C, respectively. In the 50 ns interaction of the gypsogenin molecule with CatK, it was observed that it made hydrogen bonding interactions with the ASP61 and GLY66 residues, which are located in the active binding region of the receptor and revealed in the results of molecular docking analysis. However, these interactions are limited to a shorter period of time by being covered by water molecules. Apart from these, gypsogenin was found in a much longer interaction with TYR67 and GLU115 residues with water‐interactive hydrogen bonding interactions and also performed hydrophobic interactions with LEU 209 and LEU160 residues seen in Figure 5A. The interaction between the TYR67 residue and the interacting O atom was the longest‐lasting interaction, effective 75% of the simulation time. The OH group of gypsogenin had an active interaction with the GLU115 residue for 44% of the simulation time. Another interaction was observed between the oxygen atom and the ASN208 residue, which was water interaction and effective for 29% of the simulation time.

Considering the interaction of the rutin molecule with CatK; It was seen that it formed hydrogen bonding interaction with ASP61, GLY66 and ALA163 residues. Hydrogen bonding interactions were effective between the ASP61 residue and both OH groups of the molecule, which were active for 96 and 90% of the simulation time, respectively. The hydrogen bonding interaction with ASP61 which was encountered with the result of the docking analysis before, continued to be effective here as well. ASP61 and GLY66 interactions were the most effective interactions during the 50 ns simulation, which were also revealed by molecular docking analysis. The GLY66 residue was clamped to the Oxygen atom in the interior of the molecule by hydrogen bonding for as large as 94% of the simulation time. At the same time, the internal hydrogen bonds of the OH groups of the molecule with the Oxygen atom were also observed throughout the simulation. The hydrogen bonding interaction between the ASN161 residue which was detected in the docking analysis results was restricted to water molecules and also called water bridge interaction. In the MD analysis, there was a hydrophobic interaction with the TYR67 residue and a hydrogen bonding interaction with the TRP26 residue, were determined as aromatic hydrogen bonding interaction in the docking analysis results. There was an ionic interaction between rutin and the HIS162 residue, which was expressed as hydrogen bonding interaction in the docking analysis. In addition to all these interactions, it was revealed that a hydrogen bonding interaction with ALA163 occurred with the O atom. This interaction also took place effectively in 40% of the simulation time. The types of interactions and their duration are shown in Figure 5B.

In the interaction of rosmarinic acid with CatK; It has been determined that hydrogen bond interactions occured with GLN19, GLY20, CYS25, LEU160, SER138, ASN161, GLN143, ALA137, HIS162, and TRP184 residues. The longest interactions were with GLN19, HIS162, TRP184, ASN161, and CYS25 residues. Similarly, hydrogen bonding interactions with GLN19, LEU160, HIS162, and TRP184 residues were revealed as a result of docking analysis. According to docking analysis, an aromatic hydrogen bonding and pi‐pi interactions were observed with the TYR 67 residue, while water bridge interaction together with hydrophobic interaction were revealed with the same residue in the MD calculation. Hydrophobic interactions also occurred at TRP184 and HIS162 residues. The ionic interaction that occurred with HIS162 remained effective at 55% of the simulation time. Interactions that are effective 80% of the simulation time; hydrogen bonding interaction between GLN19 and O atom, hydrogen bonding and hydrophobic hybrid interactions between HIS162 and O atom, and hybrid interactions between TRP184 and O atom. The types and durations of all these interactions are shown in Figure 5C.

The interaction of all three molecules with CatK was monitored for 50 ns simulation in the dynamic system, the stability of all three systems remained intact throughout the interactions and continued interactions with the same residues that we defined before by the docking analysis.

For the development of anti‐osteoporosis drugs, the interaction mechanisms of the CatK receptor with three of the most dominant phytochemicals in *G. eriocalyx* have been demonstrated for the first time both molecular docking analyses which occur in a vacuum environment where instantaneous interactions are effective, and also MD analyzes with dynamic systems similar to the body environment.

#### 3.2.3 ADME results

Theoretical prediction of ADME pharmacokinetic parameters is essential for efficacious and safer drugs. SwissADME web tool uses computational drug discovery methods such as BOILED‐Egg, iLOGP, and Bioavailability Radar to predict physicochemical properties, pharmacokinetics, and drug‐likeness. ADME parameters prediction results of *G. eriocalyx*'s main phytochemicals are given in Table 2. The predicted ADME properties are color‐coded to facilitate identification between different chemicals.

**TABLE 2:** Theoretical ADME properties of phytochemicals (color codes for each property are defined as pink for highly positive and yes values, yellow for weakly positive values, and green for negative and no values.

ADME properties	Gypsogenin	Rutin	Rosmarinic acid	
**Physicochemical Properties**				**Range of Drugs**
Molecular Weight (g/mol)	470.68	610.52	360.31	(50.0/500.0)
Fraction Csp3	0.87	0.44	0.11	(≥0.25)
Num. Rotatable Bonds	2	6	7	(1.0/10.0)
Num. H‐bond Acceptors	4	16	8	(2.0/20.0)
Num. H‐bond Donors	2	10	5	(0.0/6.0)
TPSA Å^2^	74.60	269.43	144.52	(≤140.0)
**Pharmacological Properties**				
Consensus Log P_o/w_	5.34	−1.29	1.52	(≥1.0, ≤4.0)
log S (Water Solubility)	−6.83	−3.30	−3.44	(−6.5/0.5)
Solubility Class	Poorly Soluble	Soluble	Soluble	Soluble
GI Absorption	High	Low	Low	‐
BBB Permeant	No	No	No	No
P‐gp Substrate	Yes	Yes	No	‐
Log Kp (Skin Permeation cm/s)	−4.43	−10.26	−6.82	(cm/h Kp)
Lipinski Rule of 5	1	3	0	(Max. 4)
Bioavailability Score	0.85	0.17	0.56	(≥0.10)

The general properties of the *G. eriocalyx*'s phytochemicals meet the molecular weight requirement of less than 500 Da for gypsogenin and rosmarinic acid. The mean of the logP values is the general expression of the lipophilicity that refers to the tendency of a compound to partition between a lipophilic organic phase and a polar aqueous phase.

High lipophilicity generally results in lower solubility, higher permeability in the gastrointestinal tract across the blood–brain barrier and other tissue membranes, and higher binding to metabolizing enzymes.^46^

Afterward, the water solubility values of the components were estimated as high and medium solubility. Values of GI absorption were found to be high for *G. eriocalyx* main component and most active ingredient, gypsogenin, and low for rutin and rosmarinic acid.

Gastrointestinal (GI) absorption, skin permeability, and P‐glycoprotein (P‐gp) substrate parameters were used to estimate the absorption level of phytochemicals. Due to the high saponin content, gastrointestinal absorption of the ingredients is expected to take a relatively long time, but to be completed successfully.^47^

Log Kp > −2.5 is considered to have low skin permeability, and based on this information, it was determined that phytochemicals have low skin permeability. The blood–brain barrier parameter is an important parameter that indicates whether the drug has any side effects on the brain. Phytochemicals were found to have no blood–brain barrier permeability.

When the drug similarities are compared according to Lipinski's rules, it can be said that GEE can show drug‐like activity when the desired properties are provided for the target region since there is no component whose number of violations exceeds four and the amount of bioavailability is sufficient.

Theoretical estimation of ADME parameters facilitates the investigation of phytochemicals in the extract as drug candidates. It was determined that the three main phytochemicals of GEE used in the study did not violate Lipinski's rule of five and could be investigated as a drug.^42^, ^48^

### 3.3 Characterization of CNPs

#### 3.3.1 Average size, polydispersity index, and zeta potential determinations

**FIGURE 6:**
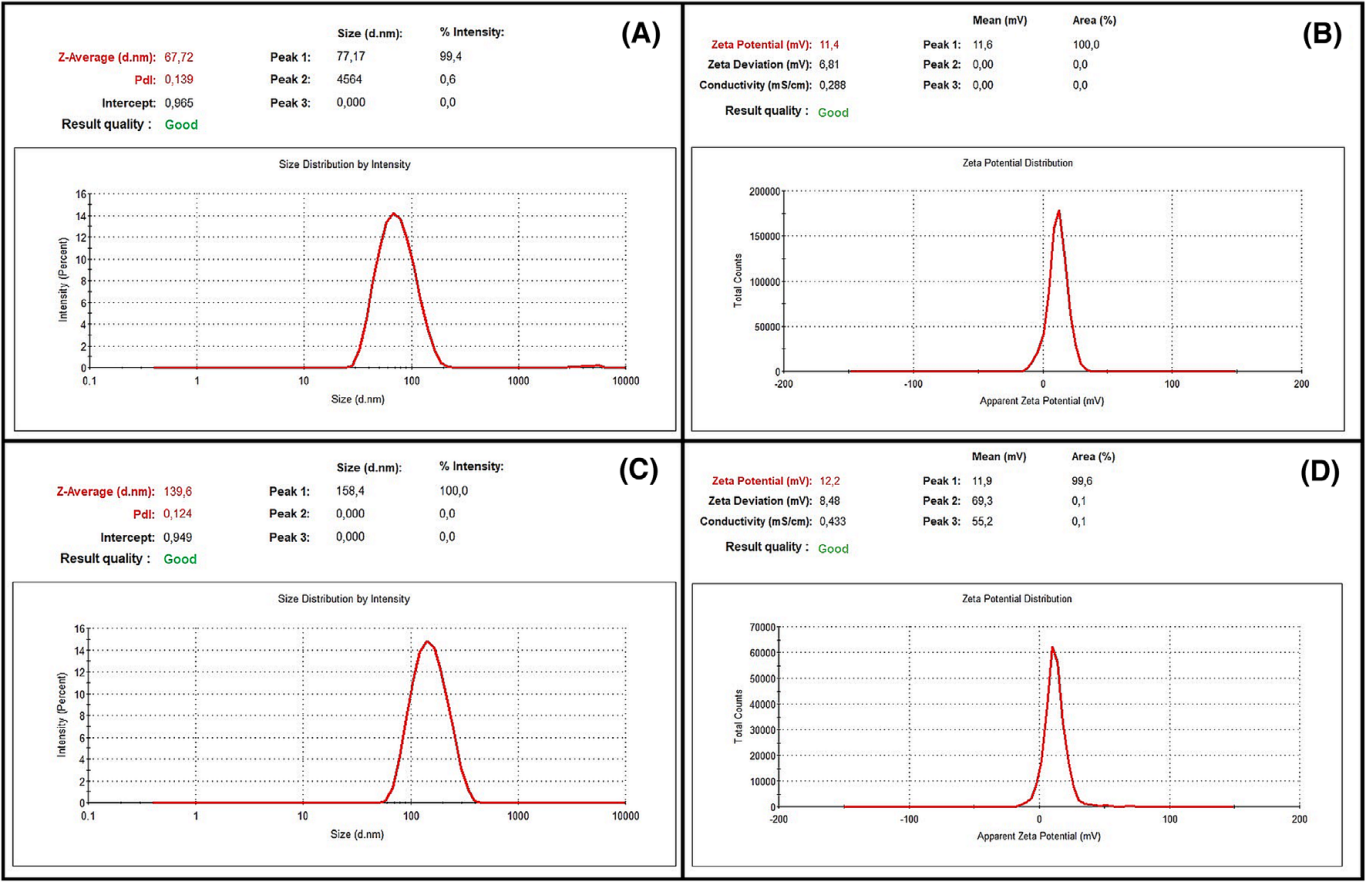
dynamic light scattering (DLS) analysis results; (A) average size and PdI value graph, and (B) zeta potential graphics of blank chitosan nanoparticles (CNPs); (C) average size and PdI value graph, (D) zeta potential graph of *Gypsophila eriocalyx* extract (GEE) loaded CNPs.

Among nanoparticle sizing methods, dynamic light scattering (DLS) has some advantages, such as easy sample preparation, fast measurement, accurate and reliable results. It has become one of the popular methods for determining the hydrodynamic size of nanoparticles.^49^ In this study polydispersity index, average size, and zeta potential of blank CNPs and GEE loaded CNPs were determined based on the DLS measurement principles. Results show that blank CNPs have an average size of 67.72 ± 7.74 nm, 0.139 ± 0.01 PdI, and 11.4 ± 1.29 mV zeta potential value (Figure 6A,B). On the other hand, GEE loaded CNPs have 139.6 ± 1.55 nm average size, 0.124 ± 0.01 PdI, and 12.2 ± 0.91 mV zeta potential (Figure 6C,D). According to the results, extract loaded CNPs increased in average particle size and zeta potential but decreased in PdI value.

Nanoparticle sizes depend on various parameters such as experimental conditions, mixing ratios, extract densities, etc.^43^ In this study, it was determined that the size of GEE‐loaded CNPs increased compared to the blank CNPs, and this increase was consistent with the studies in the literature. Rejinold et al. (2011) synthesized saponin‐loaded CNPs and analyzed their particle sizes using the DLS method. They determined that saponin‐loaded CNPs (55 ± 7 nm) increased in size compared to blank CNPs (35 ± 7 nm).^50^ Manne et al. (2020) synthesized CNPs loaded with *Pterocarpus marsupium* Roxb. heartwood extract. They stated that the nanoparticle sizes increased with the increase of the plant extract concentration, and this could be due to the interaction between the polymer and the plant extract.^51^ Interactions such as weak and/or electrostatic forces between their chemical groups may be responsible for such size changes in drug‐loaded nanoparticles.^43^, ^52^

The PdI value of 0.2 and lower indicates that the nanoparticles are more uniformly distributed.^53^ Medina‐Torres et al. (2019) synthesized CNPs loaded with bioactive phenols obtained from *Citrus latifolia* waste. They showed that these nanoparticles had a very homogeneous size distribution with a PdI value of 0.182 ± 0.06.^54^ Soltanzadeh et al. (2021) synthesized neuroprotective flavonoid‐loaded CNPs from *Phyllanthus niruri* Linn. They reported that blank CNPs had PdI values of 0.25, which indicates obtaining uniform and low‐dispersion particles.^55^ Upadhyay et al. (2023) synthesized myricetin‐loaded chitosan nanoformulations. They stated that this nanoformulation had a PdI value (0.272 ± 0.02) in the range of 0.1–0.4, representing monodispersity.^56^ Therefore, it was concluded that the blank CNPs and GEE‐loaded CNPs obtained in our study showed a uniform distribution.

It is mentioned in the literature that the plant extract loaded nanoparticles may have a higher positive value than the blank nanoparticles and this may be due to the plant extract increasing the positive charge groups on the surface of the nanoparticles.^51^ This explains the slight increase in the zeta potential for the GEE‐loaded CNPs synthesized in our study. On the other hand, it is known that cationic nanoparticles structurally support osteogenesis^57^ and intracellular uptake is higher in all cell lines because positive CNPs adapt to cell kinetics more easily.^58^ Also, positively charged nanoparticles show higher binding affinity due to the minimally negative charge of the bone surface.^59^

#### 3.3.2 TEM analysis results

**FIGURE 7:**
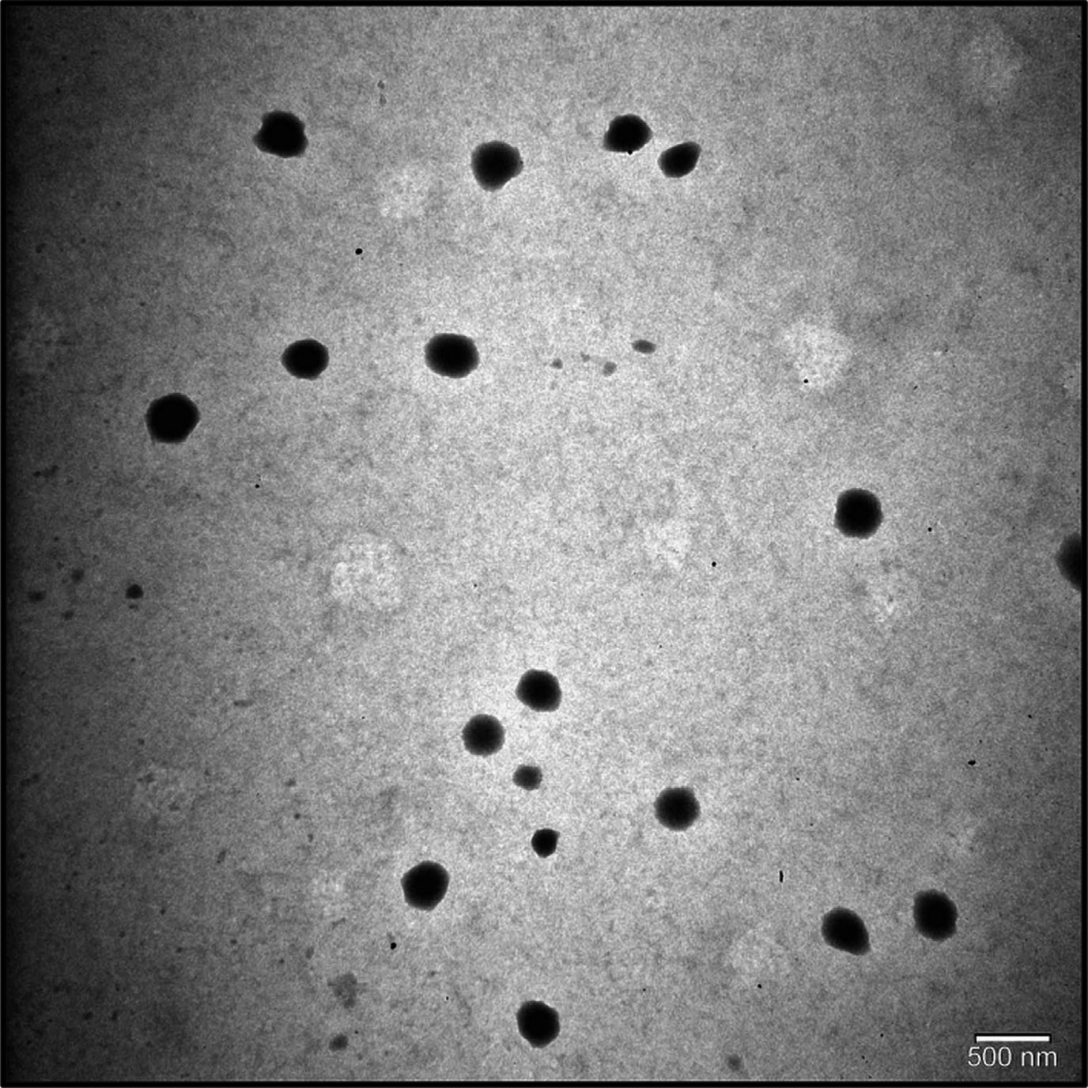
TEM analysis of *Gypsophila eriocalyx* extract (GEE) loaded chitosan nanoparticles.

The morphology of the GEE loaded CNPs was determined using TEM. The TEM image (Figure 7) confirmed that GEE loaded CNPs were homogeneously dispersed and the particles were spherical.^60^, ^61^ Similar results for plant extract‐loaded CNPs are reported in the literature. Kain and Kumar (2020) synthesized CNPs loaded with *Achillea millefolium* L. extract and determined by TEM analysis that their surface morphology was smooth and spherical in shape.^61^ Alqahtani et al. (2021) demonstrated by TEM analysis that *Jatropha pelargoniifolia* extract loaded CNPs prepared by the ionic gelation method exhibited spherical morphology and compact structure.^60^ Cansel Tiryaki et al. (2023) obtained saponin from *Cephalaria* species and synthesized saponin‐loaded CNPs using ionic gelation method. They determined by TEM analysis that empty and saponin‐loaded CNPs have varying sizes and spherical morphology.^62^ As a result, the morphological results obtained with TEM analysis in this study are similar to those of the literature.

#### 3.3.3 ATR‐FTIR analysis results

**FIGURE 8:**
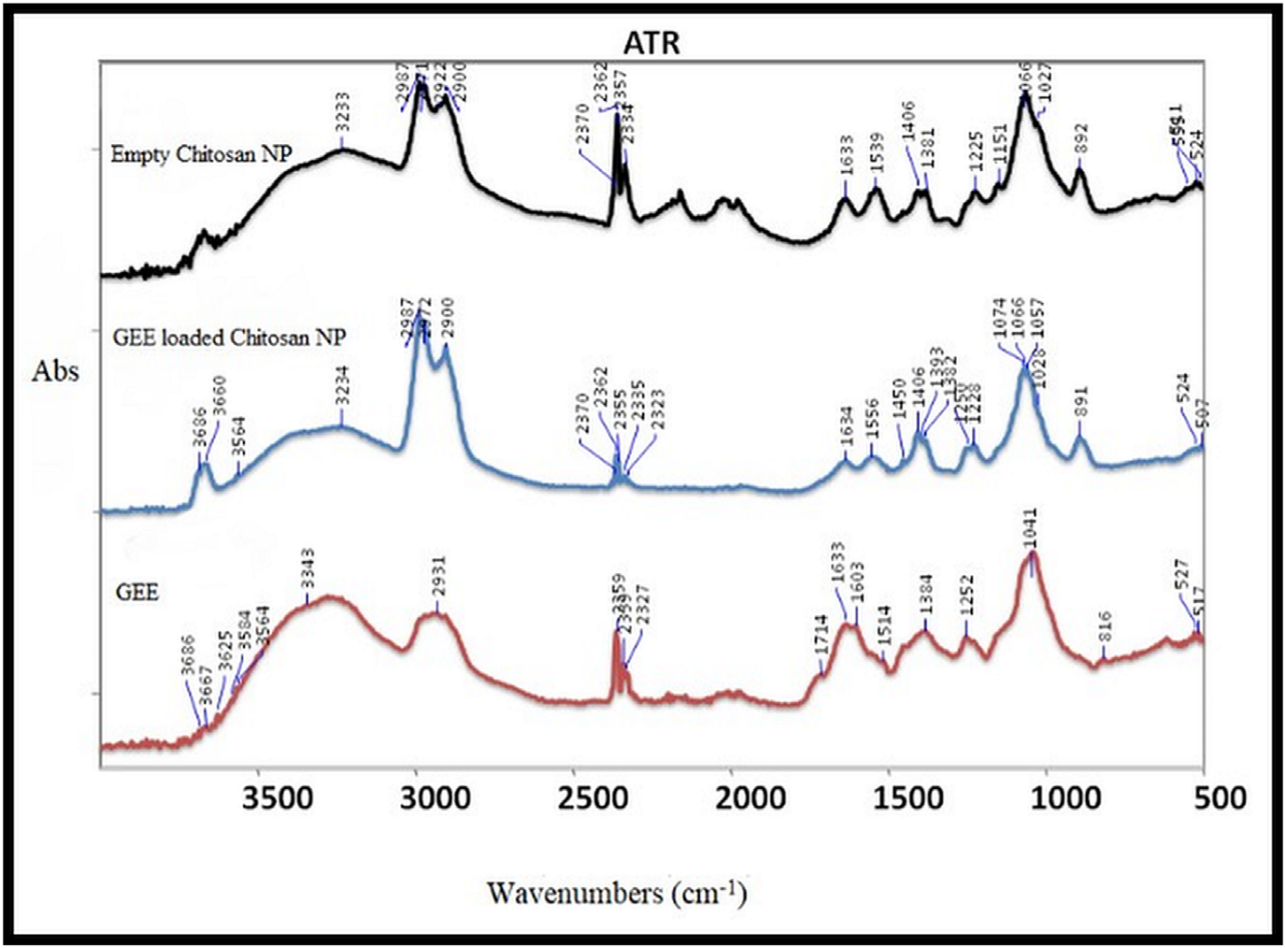
The FT‐IR spectra of blank chitosan nanoparticles, *Gypsophila eriocalyx* extract (GEE) loaded chitosan nanoparticles (CNPs) and GEE.

The FTIR spectra of blank CNPs, GEE loaded CNPs and GEE are given in Figure 8. Due to its versatile approach, ATR‐FTIR spectroscopy directly analyzes active ingredients and chemical interactions within samples. The reason why it is preferred in characterization studies is that it paves the way for the appearance, modification and distinguishability of feature peaks of various functional groups such as C=O, C‐H, or N‐H in nanoparticle formulations, especially in drug formulations. The data obtained within the scope of the study, the observation of blank chitosan NPs and GEE characteristic peaks together in GEE‐loaded CNPs indicates that the encapsulation was carried out successfully. The peaks in the range of ~3600–3000 cm^−1^ observed in the spectra described O‐H bond stretching. The peaks in the range of ~2900–2987 cm^−1^ were considered to be the CH_2_ stretching. For GEE spectrum, the C=C absorbance was observed at 1633 cm^−1^, whereas C=O absorbance was found to be at 1714 cm^−1^ indicated that the presence of saponin^63^, ^64^. In addition to this, oligosaccharide linkage absorptions to saponins, namely C‐O‐C, were given in the aqueous extract between 1046 cm^−1^ ^63^ and also observed at 1041 cm^−1^ in this study. As a result of the studies on saponin, it was concluded that the ~1714 cm^−1^ peak was also thought to be the C=O stretching band of esters, indicating the saponin content, in line with the specific peaks specified in the literature.

In another study^65^, the presence of O‐H and C‐O stretching bands of gypsum, calcium oxalate and calcium carbonate was observed in the roots, stems, leaves and flowers of *Gypsophila taxa*, which means that there is excess Ca and sulfate in the habitat where the species grow. The C‐O stretching bands corresponding to calcium carbonate were given at 1445–1390 cm^−1^.^65^ A similar peak was recorded at 1384 cm^−1^in GEE spectrum. The C‐O‐C stretching bands belonging to the ester were given 1290–1185 cm^−1^.^65^ The corresponding peak was also recorded at 1252 cm^−1^. In roots, C‐O in plane bending bands corresponding to calcium carbonate were detected at 871 cm^−1^, while the S‐O bending bands of the gypsum were also defined as 597 cm^−1^ and 669 cm^−1^. It is possible to see similar peaks in the GEE spectrum.

#### 3.3.4 Encapsulation efficiency and loading capacity

**FIGURE 9:**
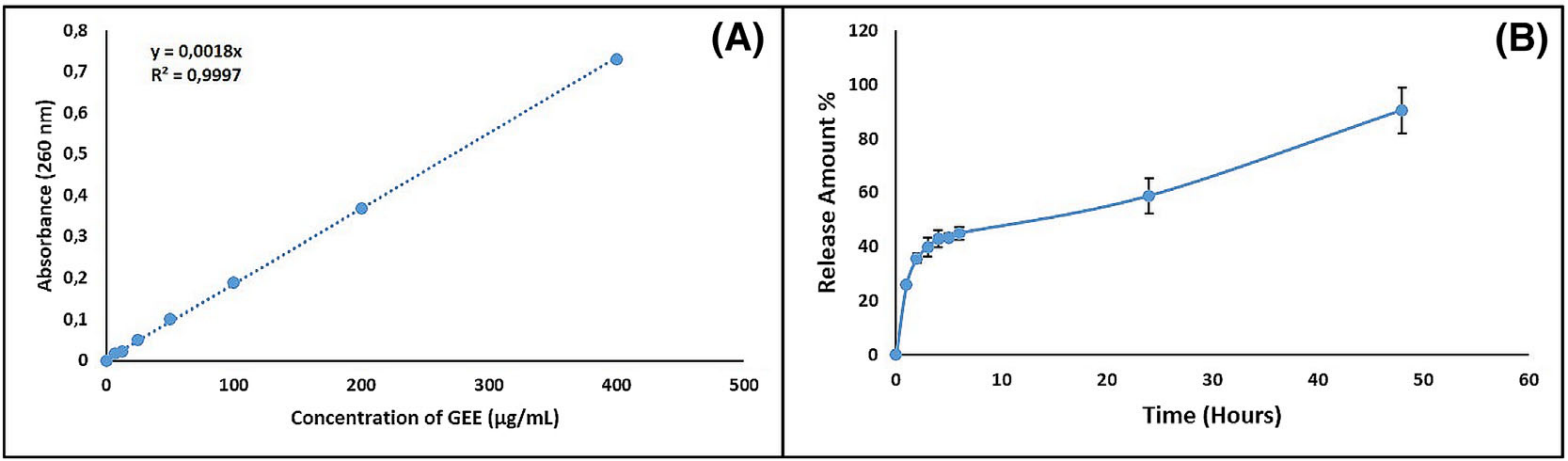
Results of in vitro release study (A) the calibration curve of *Gypsophila eriocalyx* extract (GEE), and (B) the controlled release profile of GEE (*n* = 3).

Encapsulation efficiency, which is an indicator of drug loading efficiency, is among the most important parameters in the development of nanoparticle drug delivery systems. It is very important that nanoparticles have high encapsulation efficiency and loading capacity ratios, since the size of the reservoir in which the drug molecule is loaded is extremely small compared to conventional drug delivery systems.^66^ In the study, the standard curve of GEE was prepared to determine the encapsulation efficiency and loading capacity of GEE‐loaded CNPs (Figure 9A). By using the Equation (2), encapsulation efficiency was calculated 90.61%, and loading capacity was calculated 38.56% by using Equation (3).

The encapsulation efficiency and loading capacity of GEE‐loaded CNPs synthesized in this study are higher compared to literature studies. Silva et al. (2016) synthesized rosmarinic acid‐loaded CNPs and reported an encapsulation efficiency is 60.2% and loading capacity is 5.3%.^67^ Rahimi et al. (2019) synthesized quercetin‐loaded CNPs and found that the encapsulation efficiency is 65.8% and loading capacity is 9.6%.^68^ Vitali et al. (2021) synthesized carvacrol‐loaded CNPs and determined their encapsulation efficiency and loading capacity as 56% and 25.5%, respectively.^69^ Shetta et al. (2019) synthesized peppermint oil and green tea oil loaded CNPs and determined that the loading capacities of nanoparticles were 8.15%–22.2% and 2.2%–23.1%, respectively, and the encapsulation efficiencies were 78%–82% and 22%–81%, respectively.^70^

#### 3.3.5 In vitro release study of GEE‐loaded CNPs

The release profile of GEE‐loaded CNPs was given in Figure 9B which is depending on the time and cumulative releases (%).

One of the biggest advantages of controlled drug delivery systems is that they support a slow release, rather than a single delivery of ingredients to the target site. The use of controlled drug systems in osteoporosis treatments provides a great advantage in taking individual treatment steps and adjusting the dosage.^16^ It is clear that observing a sustained release profile in the study indicates the potential for extended and controlled release in blood circulation hence retaining the substance until desired osteoporotic environment. Depending on the in vitro release study results, 90.61% of GEE was released within 48 h. In the first 10 h, 40% of the drug was released rapidly. This release is called a “bolus” dose and is often preferred in therapeutic applications of controlled drug delivery systems.^71^ It allows about half of the drug to reach the target area quickly, which provides relief in the target area without waiting for the full release to occur.^72^ In controlled release formulations, an initial bolus of a drug is released ahead of the release profile reaching a stable state. This phenomenon is named ‘burst release’ and leads to higher initial drug delivery. Initial burst release provides immediate relief in the target area then followed by controlled release of the drug.^73^ The same release trend was observed in a study of alendronate sodium encapsulated PLGA microspheres, which are used in the treatment of osteoporosis. Results suggest that 70% of the drugs are released within 48 h.^74^ In the release profiles experiment of CNPs loaded with GEE, it was observed that approximately 90.45% of the encapsulated GEE amount was released into the PBS environment after 48 h. In line with the results obtained, it was found that 40% of the nano‐formulation was released rapidly within the first 10 h. In this context, it was concluded that GEE‐loaded CNPs show a sustained release profile and therefore have a long‐term and controlled release potential in the bloodstream, and the extract can be retained up to the desired osteoporotic environment.

### 3.4 MTT assay results

#### 3.4.1 Cytotoxicity results

**FIGURE 10:**
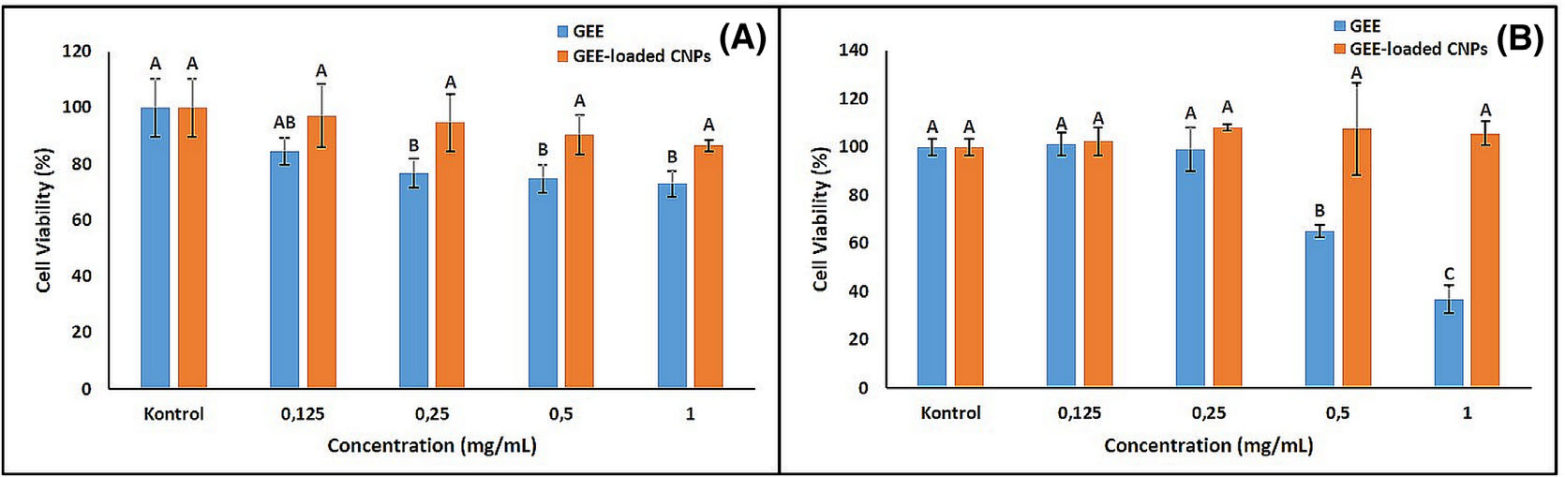
Results of in vitro cytotoxicy and osteogenic activity studies (A) cytotoxicity of *Gypsophila eriocalyx* extract (GEE) and GEE‐loaded chitosan nanoparticles (CNPs), and (B) osteogenic proliferation of GEE and GEE‐loaded CNPs.

Evaluation of the cytotoxicity of nanocarriers is an important parameter when developing a drug delivery system. The biochemical properties of nanoparticles provide insight into cellular cytotoxicity using various cell models.^75^ Among these cell models, the L929 fibroblast cell line is widely used in cytotoxicity tests for cellular viability and proliferation due to its high proliferative nature and features of fibroblast cells, such as extracellular collagen matrix synthesis and regulating neighboring cell behavior.^76^ Moreover, the applicability of L929 in in vitro cytotoxicity studies is recommended by international standards such as ISO 10993‐5.^77^ On the other hand, the MTT test is an important calorimetric test that determines the viability of cells when exposed to toxic substances.^78^, ^79^ Therefore, the cytotoxicity of GEE‐loaded CNPs was evaluated on L929 cells by MTT assay. Results of in vitro cytotoxicity analysis for GEE and GEE‐loaded CNPs are given in Table 3 and Figure 10A.

**TABLE 3:** In vitro cytotoxicity analysis results (values are given as mean ± SD).

GEE		GEE‐loaded CNPs	
Concentration (mg/mL)	Cell viability (%)	Concentration (mg/mL)	Cell viability (%)
0	100.00 ± 10.32^A^	0	100.00 ± 10.32^A^
0.125	84.37 ± 4.82^AB^	0.125	97.19 ± 11.10^A^
0.25	76.82 ± 5.14^B^	0.25	94.65 ± 10.14^A^
0.5	74.79 ± 5.03^B^	0.5	90.30 ± 7.01^A^
1	73.00 ± 4.64^B^	1	86.55 ± 1.96^A^

*Note*: Different letters indicate statistically significant differences (*P* ≤ 0.05).

In line with ISO10993 (International Standards Organization) standards, it has been stated that the substances are non‐toxic at a cell vitality level above 80%.^80^ As given in Figure 10A, cell viability was below 80% at 0.25, 0.5, and 1 mg/mL GEE concentrations, and these decreases were determined to be statistically significant (*p* < 0.05). However, no high toxic effects were observed at any sample concentrations of GEE‐loaded CNPs, and cell viability remained above 80% even at the highest concentration. In this context, it was determined that GEE‐loaded CNPs had no cytotoxic effect at relevant concentrations, and this nanoformulation was less toxic than GEE. Rejinold et al. (2011) showed that saponin‐loaded CNPs were not toxic in the 0.1‐ 1.0‐mg/mL concentration range on L929 and NIH‐3T3 cell lines.^50^ De Oliveira et al. (2021) showed on the L929 cell line that CNPs loaded with *Baccharis dracunculifolia* leaf bud extract did not change cell viability.^81^ Moreover, Servat‐Medina et al. (2015) synthesized CNPs loaded with *Arrabidaea chica* extract and examined their effects on human skin fibroblasts by MTT assay. They reported that cell viability for the standard extract applied at a concentration of 0.5 mg/ml was 30% compared to the control. However, this effect was not observed in standard extract‐loaded CNPs.^82^ Mi et al. (2021) synthesized CNPs containing adriamycin using negatively charged carboxymethyl chitosan and positively charged 2‐hydroxypropyltrimethyl ammonium chloride chitosan. They showed that free adriamycin reduced the viability of normal L929 cells by 20.15% at 1000 μg/mL, but the cytotoxic effect was reduced by loading this substance into CNPs.^83^ In this context, it was concluded that the lower toxicity of GEE‐loaded CNPs compared to GEE was due to biofunctionalization with chitosan.

#### 3.4.2 Osteogenic proliferation analysis results

To determine the effect of GEE and GEE‐loaded CNPs on osteogenesis, their contribution to osteogenic proliferation in human bone marrow stem cells (hBMC) *in vitro* was examined comparatively. The results were given in Table 4 and Figure 10B. According to the osteogenic proliferation analysis, a 1.19% cell proliferation enhancing effect of 0.125 mg/mL GEE concentration on hBMCs was determined compared to the control group. However, it was determined that 0.5 and 1 mg/mL concentrations of GEE showed 34.95% and 63.17% toxicity on the cells, respectively, and this effect was statistically significant (*p* < 0.05). In addition, it was determined that cell proliferation increased at all concentrations of GEE‐loaded CNPs used in the experiment. Controlled release of GEE by encapsulating it with chitosan has demonstrated that the formulation is biocompatible and has a cell proliferation‐enhancing behavior at every concentration applied at the nanoscale.

**TABLE 4:** Osteogenic proliferation analysis results (values are given as mean ± SD).

GEE		GEE‐loaded CNPs	
Concentration (mg/mL)	Cell viability (%)	Concentration (mg/mL)	Cell viability (%)
0	100.00 ± 3.56^A^	0	100.00 ± 3.56^A^
0.125	101.19 ± 4.75^A^	0.125	102.45 ± 5.76^A^
0.25	99.15 ± 8.88^A^	0.25	108.10 ± 1.10^A^
0.5	65.05 ± 2.55^B^	0.5	107.47 ± 18.95^A^
1	36.83 ± 5.81^C^	1	105.71 ± 5.06^A^

*Note*: Different letters indicate statistically significant differences (*P* ≤ 0.05).

GEE had osteogenic proliferation‐enhancing effects on hBMCs at a concentration of 0.125 mg/mL, but concentrations of 0.5 and 1 mg/mL significantly reduced cell viability. This situation was examined through studies in the literature. The effects of 1–100 μg/mL concentrations of licorice extracts on human bone marrow mesenchymal stem cells (hBM‐MSCs) cell proliferation were evaluated by MTT test. It was reported that cell proliferation increased significantly at concentrations of 10, 25, and 50 μg/mL, but 100‐μg/mL concentration had a toxic effect on the cells and inhibited their proliferation.^84^ In another study, an MTT assay was applied to examine the cytotoxic effects of *Tithonia diversifolia* leaf extract on mouse bone marrow stem cells. As a result of the study, it was stated that the plant extract killed 50% of the cells at concentrations of 0.5, 0.25, and 0.125 mg/mL, and cell viability was 72% at a concentration of only 0.0625 mg/mL.^85^ In this context, it was concluded that the relevant extract concentrations were toxic to hBMCs.

On the other hand, GEE‐loaded CNPs were determined to have osteogenic proliferation‐enhancing effects on hBMCs in the concentration range of 0.125–1 mg/mL. In this context, this difference seen between GEE and GEE‐loaded CNPs was evaluated through other studies in the literature. In a study examining the effects of naringin‐loaded CNPs on bone regeneration, it was reported that the application of 20–100 μM naringin increased cell viability in Vero cells depending on the concentration, and this effect was further strengthened by the addition of naringin‐loaded CNPs to 100 μM naringin. Additionally, maximum cell viability was observed as a result of the application of both naringin and naringin‐loaded CNPs.^86^ Moreover, it is known that CNPs have a proliferative effect on bone marrow stem cells.^87^ In a study, the osteo inductive potentials of various nanoparticle formulations, including chitosan/hydroxyapatite nanoparticles, were investigated.^88^ As a result, nanoparticles did not show significant cytotoxicity at different concentrations and were reported to support proliferation limitedly. In this context, it was thought that nanoparticles synthesized with chitosan biopolymer contributed to increasing the osteogenic proliferation effect of GEE.

## 4 CONCLUSION

Gypsophila species have great pharmacological potentials such as antimicrobial, anticancer, and antioxidant. Furthermore, it was known that saponin contents of different species may have anti‐osteoporotic effects, which is the main component of Gypsophila species. In this study, GEE‐loaded CNPs were successfully synthesized for the development of a controlled drug delivery system for the treatment of osteoporosis. *In vitro* osteogenic proliferation analysis and in silico analysis were performed to investigate the effects of GEE on bone cells and interaction mechanisms. It was determined that GEE‐loaded CNPs have a long‐term and controlled release profile and can ensure the uptake of the extract into the osteoporotic environment. Different sample concentrations used in the experiments did not show cytotoxic effects, and GEE‐loaded CNPs exhibited lower toxicity than GEE. It was found by in silico molecular docking method that GEE phytochemicals have electrostatic and also hydrophobic interactions. Under ideal MD conditions, the most important thing to consider is to obtain an inhibition behavior that does not deviate from the initial structure, has a high binding affinity as much as possible, and is clamped to the target receptor from different points. During the MD analysis, no breaks or atomic changes were recorded in either the phytochemicals or the bonds in the receptor. In addition, it has been determined that the three main phytochemicals in the content of GEE are not against Lipinski's five rules and can be investigated as drugs. In conclusion, the aim of this study is to contribute to the design of a plant‐based controlled release system for use in the treatment of osteoporosis. According to the results obtained, it has been shown that GEE‐loaded CNPs may be a useful approach for the treatment of osteoporosis.

## Data Availability

Data will be made available on request.
